# Mental health and well-being trends among children and young people in the UK, 1995–2014: analysis of repeated cross-sectional national health surveys

**DOI:** 10.1017/S0033291718001757

**Published:** 2018-09-11

**Authors:** Jacqueline Pitchforth, Katie Fahy, Tamsin Ford, Miranda Wolpert, Russell M. Viner, Dougal S. Hargreaves

**Affiliations:** 1 Population, Policy and Practice Programme, UCL Great Ormond Street Institute of Child Health, London, WC1N 1EH, UK; 2 University of Exeter Medical School, Heavitree Road, Exeter EX1 2LU, UK; 3 Anna Freud National Centre for Children and Families, 12 Maresfield Gardens, London, NW3 5SU, UK; 4 Nuffield Trust, London, UK

**Keywords:** Child and adolescent mental health, epidemiology, time trends

## Abstract

**Background:**

There is a growing concern about the mental health of children and young people (CYP) in the UK, with increasing demand for counselling services, admissions for self-harm and referrals to mental health services. We investigated whether there have been similar recent trends in selected mental health outcomes among CYP in national health surveys from England, Scotland and Wales.

**Methods:**

Data were analysed from 140 830 participants (4–24 years, stratified into 4–12, 13–15, 16–24 years) in 36 national surveys in England, Scotland and Wales, 1995–2014. Regression models were used to examine time trends in seven parent/self-reported variables: general health, any long-standing health condition, long-standing mental health condition; Warwick–Edinburgh Mental Wellbeing Score (WEMWBS), above-threshold Strengths and Difficulties Questionnaire Total (SDQT) score, SDQ Emotion (SDQE) score, General Health Questionnaire (GHQ) score.

**Results:**

Across all participants aged 4–24, long-standing mental health conditions increased in England (0.8–4.8% over 19 years), Scotland (2.3–6.0%, 11 years) and Wales (2.6–4.1%, 7 years) (all *p* < 0.001). Among young children (4–12 years), the proportion reporting high SDQT and SDQE scores decreased significantly among both boys and girls in England [SDQE: odds ratio (OR) 0.97 (0.96–0.98), *p* < 0.001] and girls in Scotland [SDQE: OR 0.96 (0.93–0.99), *p* = 0.005]. The proportion with high SDQE scores (13–15 years) decreased in England [OR 0.98 (0.96–0.99), *p* = 0.006] but increased in Wales [OR 1.07 (1.03–1.10), *p* < 0.001]. The proportion with high GHQ scores decreased among English women (16–24 years) [OR 0.98 (0.98–0.99), *p* = 0.002].

**Conclusions:**

Despite a striking increase in the reported prevalence of long-standing mental health conditions among UK CYP, there was relatively little change in questionnaire scores reflecting psychological distress and emotional well-being.

What is already known on this subject?

• There is a growing concern about the mental health and well-being of children and young people (CYP) in the UK, with increasing demand for counselling services, hospital admissions for self-harm and referrals to specialist Child and Adolescent Mental Health Services.
• Few national data on trends or the prevalence of psychological distress or mental health conditions among CYP have been published in the UK since 2007.

What this study adds

• Our study found a striking increase in the reported prevalence of long-standing mental health conditions since 1995.
• There was no consistent increase in reported psychological distress among CYP over the last two decades, when measured using scores in validated questionnaires.
• However, there was some evidence of worsening trends in psychological distress and well-being of young adults in recent years (since 2011).

## Introduction

There has been a growing global concern about the mental health and well-being of children and young people (CYP) (0–24 years). Increasing rates of anxiety and depression have been reported among adolescents and young adults in a number of developed countries, 1990–2010, although wide variation exists between different countries (Murray *et al*., 2012; Bor *et al*., 2014; Collishaw, 2015). In the UK, demand for counselling services, hospital admissions for self-harm and referrals to specialist Child and Adolescent Mental Health Services (CAMHS) have all increased significantly for this age group in recent years (Hagell *et al*., 2015; National Society for Prevention of Cruelty to Children, 2016; Page, 2016; Yeung *et al*., 2016). There is also some evidence from survey data of decreases in well-being and increases in emotional problems among teenage girls (Brooks *et al*., 2015; Fink *et al*., 2015) and mental health disorders in young adult women (Pym, 2016).

Many factors have been proposed as potentially contributing to increasing mental health problems in this age group. These include social and economic changes leading to a lengthening of dependence on parents and delay in reaching more adult levels of autonomy (Patton *et al*., 2016), effects of social media (Bell *et al*., 2015), the penetration of cyberbullying into all parts of young people's lives (Bottino *et al*., 2015; National Children’s Bureau and Association of School and College Leaders, 2016) and a more highly pressurised school culture (Lessof *et al*., 2016). More recently, the global financial crisis of 2008 has been linked to an increase in suicide and wider mental health problems among both young and older adults in Greece and other European countries (Divya *et al*., 2016).

The need to understand and meet the mental health needs of CYP is reinforced by growing recognition of the importance of early life for lifetime mental health problems. Three-quarters of life-time mental health problems (excluding dementia) occur by age 24 years (Kessler *et al*., 2005). High levels of unmet need for mental health services among CYP exist in most localities in the UK (McShane and Rouse, 2015). Failure to meet these needs may have economic as well as personal and social costs, as there is good evidence that early access to appropriate health services has a potential to improve outcomes of both mild/moderate and more serious mental health problems (Marshall *et al*., 2005; Hagell *et al*., 2015; Hargreaves *et al*., 2015; National Institute for Health and Care Excellence (NICE) and NHS England, 2016).

Robust, national-level data on the prevalence and patterns of mental health problems among CYP are needed for planning and improving clinical services, understanding societal influences on mental health and evaluating the impact of different policies for CYP in UK countries. However, no detailed national studies have been done that specifically investigate trends in the mental health of CYP (0–24 years) in the UK since the studies performed in 2004 and 2007 (Green *et al*., 2005; Parry-Langdon, 2008). To address this gap, we aimed to analyse trends in mental health outcomes among CYP in national health surveys from England, Scotland and Wales over the last two decades. We also included data on total long-standing conditions (physical and mental health) and general health as mental health is a major component of each of these.

## Methods

### Design

Retrospective, secondary analysis of data from nationally representative, cross-sectional, repeated (mostly annual) surveys in the UK. Data were analysed from 140 830 participants (4–24 years) in 36 national surveys in England, Scotland and Wales, 1995–2014.

### Data

All national health surveys conducted since 1995 in England, Scotland, Wales or Northern Ireland were accessed. These are repeated, household-based, surveys that use stratified random sampling of individuals from a sample of postcode sectors each year to collect nationally representative data using consistent methodology (Darlington *et al*., 2014; Scottish_Government; Welsh_Government). The documentation of each survey was searched for all mental and physical health variables applicable to participants aged 0–24 years. At this stage, Northern Ireland was excluded as only one survey in 2007 contained relevant data, so trend analysis was not possible. Children under the age of 4 years were also excluded as no mental health variables were collected in this age group, with the exception of parent-reported long-standing mental health condition, which was very rare. For all measures, parents responded on behalf of children aged 4–12 years. Participants aged 13–15 years were invited to complete the survey themselves in England and Wales, while in Scotland, either parents or young people could complete the survey in this age group. In all countries, young people aged 16 years or over responded for themselves.

Table 1 presents the mental and physical health variables that were available for analysis by age, year and country. Data on 140 830 participants 4–24 years were analysed from the Health Survey for England (HSE) (18 surveys between 1995 and 2014, *N* = 92 422, 49.4% male), Scottish Health Survey (SHS) (eight surveys 2003–2014, *N* = 16 862, 49.0% male) and Welsh Health Survey (WHS) (eight surveys 2007–2014, *N* = 31 546, 49.9% male). See Appendix Table A for numbers of males and females in each survey. Response and missingness rates have been published previously and are fairly consistent over time and between countries. For example, in the HSE 2014, individual response rates were 63% (0–15 years) and 55% (16+ years); missingness rates were <0.5% for general health, long-standing illness and mental health condition; 2.9% for WEMWBS; and 13.0% for GHQ. Due to these low rates of missingness, missing data were not imputed. All data are publically available and were downloaded through the UK Data Service https://www.ukdataservice.ac.uk/. Data were merged and cleaned for time-series analysis following previously published guidance (Darlington *et al*., 2014).

**Table 1.** Available mental health variables and selected physical health variables by country, age and year

Health Survey for England		
	Age group	Year
Self-/parent-reported general health	4–24	1996–2014
Long-standing condition	4–24	1995–2009 and 2011–2014
Long-standing mental health condition	4–24	1995–2009 and 2011–2014
Strengths and Difficulties Questionnaire. Total (SDQT) and emotional (SDQE) scales	4–15	1997, 1999, 2002, 2004–2008, 2010–2011
General Health Questionnaire (GHQ)	13–24	1995, 1997–2006, 2008–2010, 2012, 2014
Warwick–Edinburgh Mental Wellbeing Scale (WEMWBS)	16–24	2010–2014
Scottish Health Survey		
	Age group	Year
Self-/parent-reported general health	4–24	2003, 2008–2014
Long-standing condition	4–24	2003, 2008–2014
Long-standing mental health condition	4–24	2003, 2008–2014
SDQT	4–12	2003, 2008–2009, 2011–2014
SDQE	4–12	2003, 2008–2014
GHQ	13–24	2003, 2008–2014
WEMWBS	13–15	2012–2014
WEMWBS	16–24	2008–2014
Welsh Health Survey		
	Age group	Year
Self-/parent-reported general health	4–24	2007–2014
Long-standing condition	4–15	2007–2014
Currently treated for chronic condition	16–24	2007–2014
Currently treated for mental health condition	4–24	2007–2014
SDQT/SDQE	4–15	2007–2014

### Outcomes

#### General health (physical and mental)

– Self-/parent-reported health: dichotomised as very good/good *v*. fair/bad/very bad;
– Note that Welsh response options for adults were excellent/very good/good *v*. fair/poor;
– Presence of any long-standing condition: yes/no (England and Scotland only). In Wales, a similar but differently worded question was asked (presence of any currently treated chronic illness: yes/no).

#### Mental health

– Presence of any long-standing mental health condition: yes/no (England and Scotland only). Please note that long-standing mental health conditions are a subset of those who reported ‘any long-standing condition’. Depending on the survey and year, participants may have either chosen the category ‘mental health’ from a list of long-standing conditions or given an associated free-text response. Note that the ‘mental health’ category does not include problems related to memory, learning/understanding/concentrating or social/behavioural problems (examples given in the HSE are autism/attention-deficit disorder/Asperger's syndrome). Also note that in Wales, a slightly different question was asked;
- Presence of any currently treated mental health problem: yes/no;
- Strengths and Difficulties Questionnaire Total difficulties (SDQT) score (Goodman *et al*., 2000).

Both the total score and a dichotomised score of 0–16 *v*. 17–40 were analysed.

– Strengths and Difficulties Questionnaire Emotional difficulties (SDQE) score (Goodman *et al*., 2000): the full score and a dichotomised score of 0–4 *v*. 5–10 were analysed. We investigated the SDQE score separately to the total SDQ as previous studies have raised specific concerns about increasing rates of emotional distress, especially among adolescent girls (Fink *et al*., 2015). Detailed analysis of other subscales was beyond the scope of this study. For both SDQT and SDQE, we used thresholds previously published for analysis of parent-reported SDQ scores for children aged 4–17 years (Youth_in_Mind, 2016). Individuals who scored above the described threshold were considered to have screened positive for significant mental health symptoms.
– General Health Questionnaire (GHQ) score (Goldberg *et al*., 1997): the total score and a dichotomised score (0–3 *v*. 4–12) were analysed, following previous analysis of GHQ scores in the HSE (Knott).
– Warwick–Edinburgh Mental Wellbeing Scores (WEMWBS) (Clarke *et al*., 2011). No standard threshold has been published, so only the total score was analysed.

The wording of the questions and responses in each questionnaire is presented in Appendix Table B.

### Analysis

For consistency between countries, the data were analysed in three age bands: children 4–12 years, early adolescents 13–15 years and young people 16–24 years. We limited ourselves to descriptive analyses of differences between countries and did not formally test these due to differences in survey methodology. Within each country, the proportion of males and females reporting each dichotomous variable was compared.

For each country, unweighted logistic regression models were performed for each dichotomous variable on time (in years). Similarly, unweighted linear regression models were performed for each scale variable on time (in years) for each country. Quadratic time function was included in each model where it was statistically significant (*p* < 0.05). No other covariates were included. Models were performed for all participants and then repeated for males and females separately. Some datasets included weights based on either sampling probability or a combination of sampling probability and non-response; for England, weighting data for adults were only available from 2002 onwards. Sensitivity analyses were performed comparing the outputs of regression models with weighted and unweighted data.

Additionally, sensitivity analyses were performed to investigate the recent trends from 2011 to 2014.

Due to the large number of comparisons, a *p* value of <0.01 was considered significant. All analyses were performed using SPSS version 22.

### Ethics

No ethics review was needed for these secondary analyses of publically available, anonymised data. The authors assert that all procedures contributing to this work comply with the ethical standards of the relevant national and institutional committees on human experimentation and with the Helsinki Declaration of 1975, as revised in 2008.

## Results

**Fig. 1.**
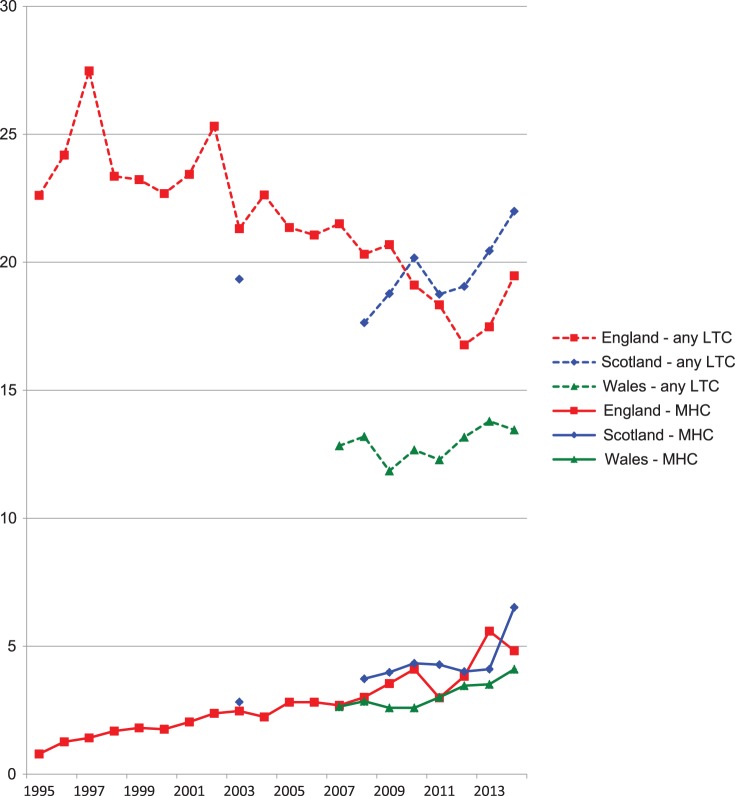
Self-/parent-reported prevalence of mental health condition and any long-term condition, 4–24 years, UK countries, 1995–2014. Source: Health Survey for England (HSE), Scottish Health Survey (SHS), Welsh Health Survey (WHS). LTC, long-term condition; MHC, mental health condition. Note that wording differs between questionnaires – see Methods section and Appendix for details.

There was a consistent increase in long-standing mental health conditions over time (see Fig. 1). Across all participants aged 4–24 years, the prevalence increased sixfold over the 19-year period in England (from 0.8% to 4.8%, *p* < 0.001), more than doubled in Scotland over 11 years (2.8–6.5%, *p* < 0.001) and increased by more than half in Wales over 7 years (2.6–4.1%, *p* < 0.001). Over the corresponding time periods, the prevalence of total long-standing conditions decreased slightly in England (22.6–19.5%, *p* < 0.001), increased slightly in Scotland (19.3–22.0%, *p* < 0.001) and was unchanged in Wales (12.8% *v*. 13.5%, *p* = 0.10).

The prevalence of each health problem by country, age and sex is summarised below and presented in Appendix Table C. Findings from the regression models for each country are presented in Tables 2–4. All tables show unweighted data. Repeating the analyses with weighting (where available) had minimal impact on these findings (see Appendix Table D).

**Table 2.** Odds ratios for changes in mental health and well-being measures over time, by age group and sex. Scottish Health Survey, 2003–2014

	Total				Males				Females			
	Odds ratio/*β* coefficient	Confidence interval		*p* value	Odds ratio/*β* coefficient	Confidence interval		*p* value	Odds ratio/*β* coefficient	Confidence interval		*p* value
	4–12 years old											
Parent-reported general health	0.97	0.94	0.99	0.010	0.97	0.94	1.00	0.085	0.96	0.93	1.00	0.054
Mental health condition	1.05	1.02	1.08	0.004	1.07	1.03	1.11	0.001	1.00	0.94	1.07	0.908
Long-standing illness	0.99	0.98	1.00	0.143	0.97	0.96	0.99	0.008	1.01	0.99	1.03	0.447
SDQE binary	0.98	0.96	1.00	0.079	1.00	0.98	1.03	0.764	0.96	0.93	0.99	0.005
SDQE	−0.01	−0.02	0.00	0.011	0.00	−0.01	0.02	0.699	−0.03	−0.04	−0.02	<0.001
SDQT binary	0.99	0.97	1.01	0.328	1.01	0.98	1.04	0.455	0.95	0.92	0.99	0.009
SDQT	−0.05	−0.08	−0.02	0.002	−0.01	−0.05	0.04	0.818	−0.09	−0.14	−0.05	<0.001
	13–15 years old											
Parent-reported general health	0.99	0.95	1.03	0.480	0.98	0.93	1.03	0.433	0.99	0.94	1.05	0.839
Mental health condition	1.07	1.01	1.13	0.024	1.04	0.98	1.12	0.197	1.11	1.00	1.24	0.045
Long-standing illness	1.03	1.01	1.06	0.015	1.04	1.01	1.08	0.023	1.02	0.98	1.06	0.277
GHQ binary	1.02	0.99	1.06	0.200	1.05	0.98	1.11	0.145	1.01	0.97	1.06	0.563
GHQ	0.02	0.00	0.04	0.056	0.02	0.00	0.04	0.078	0.02	−0.01	0.05	0.238
WEMWBS	−0.45	−1.12	0.23	0.192	0.10	−0.79	0.99	0.829	−1.06	−2.07	−0.05	0.039
	16–24 years old											
Self-reported general health	1.00	0.97	1.03	0.916	0.99	0.95	1.03	0.637	1.00	0.97	1.04	0.785
Mental health condition	1.08	1.03	1.13	<0.001	1.07	1.00	1.15	0.044	1.08	1.03	1.14	0.003
Long-standing illness	1.04	1.01	1.06	0.001	1.02	0.98	1.05	0.375	1.05	1.02	1.08	0.001
GHQ binary	1.02	1.00	1.05	0.083	1.01	0.97	1.06	0.574	1.03	1.00	1.06	0.071
GHQ	0.03	0.01	0.05	0.011	0.01	−0.02	0.04	0.495	0.05	0.01	0.08	0.006
WEMWBS	−0.13	−0.27	0.01	0.072	−0.06	−0.26	0.15	0.604	−0.21	−0.40	−0.02	0.033

SDQT, Strengths and Difficulties Questionnaire Total Score (binary score 0–16 *v*. 17–36); SDQE, Strengths and Difficulties Questionnaire Emotion Score (binary score 0–4 *v*. 5–10); GHQ, General Health Questionnaire (binary score 0–3 *v*. 4–12); WEMWBS, Warwick–Edinburgh Mental Health and Wellbeing Score.

For all binary variables, odds ratios show the change in odds per year over the study period. Values >1 indicate increasing odds of poor health over time. For scale variables (SDQE, SDQT, WEMWBS), the *β* coefficient represents the change in score per year over time. Positive values indicate increasing number of symptoms (SDQT, SDQE) or increasing well-being score (WEMWBS).

**Table 3.** Odds ratios for changes in mental health and well-being measures over time, by age group and sex. Welsh Health Survey, 2007–2014

	Total				Males				Females			
	Odds ratio/*β* coefficient	Confidence interval		*p* value	Odds ratio/*β* coefficient	Confidence interval		*p* value	Odds ratio/*β* coefficient	Confidence interval		*p* value
	4–12 years old											
Parent-reported general health	1.00	0.93	1.07	0.979	1.03	0.94	1.13	0.499	0.95	0.86	1.06	0.382
Parent-reported general health	0.97	0.94	1.01	0.137	0.97	0.93	1.02	0.247	0.97	0.92	1.03	0.324
Mental health condition	0.96	0.89	1.03	0.228	0.92	0.85	1.01	0.086	1.02	0.90	1.16	0.760
Any limiting long-standing illness	1.00	0.97	1.04	0.819	1.00	0.96	1.04	0.827	1.01	0.97	1.07	0.586
Long-standing illness	1.01	0.99	1.02	0.582	0.99	0.96	1.01	0.352	1.03	1.00	1.06	0.078
SDQE binary	1.01	0.98	1.04	0.567	1.01	0.97	1.05	0.759	1.00	0.97	1.04	0.816
SDQE	−0.01	−0.02	0.01	0.261	−0.01	−0.04	0.01	0.167	0.00	−0.02	0.03	0.644
SDQT binary	0.98	0.95	1.01	0.209	0.98	0.94	1.01	0.228	0.98	0.94	1.03	0.518
SDQT	−0.04	−0.08	0.01	0.115	−0.07	−0.14	−0.01	0.031	0.03	−0.03	0.09	0.372
	13–15 years old											
Self-reported general health	0.97	0.87	1.07	0.541	1.00	0.86	1.17	0.990	0.94	0.81	1.08	0.382
Self-reported general health	0.96	0.92	1.01	0.114	0.97	0.91	1.04	0.371	0.96	0.89	1.02	0.175
Mental health condition	1.04	0.95	1.14	0.376	1.01	0.89	1.14	0.873	1.08	0.95	1.24	0.247
Any limiting long-standing illness	1.00	0.95	1.05	0.908	1.05	0.99	1.12	0.107	0.93	0.87	1.00	0.053
Long-standing illness	0.97	0.94	1.00	0.073	0.98	0.94	1.02	0.358	0.96	0.92	1.01	0.111
SDQE binary	1.07	1.03	1.10	<0.001	1.05	0.99	1.11	0.109	1.07	1.02	1.12	0.002
SDQE	0.05	0.02	0.08	<0.001	0.02	−0.02	0.05	0.282	0.08	0.03	0.12	0.001
SDQT binary	1.01	0.97	1.05	0.590	0.99	0.94	1.05	0.713	1.03	0.98	1.09	0.266
SDQT	0.03	−0.05	0.11	0.497	0.00	−0.11	0.11	0.963	0.10	−0.01	0.21	0.074
	16–24 years old											
Self-reported general health	1.00	0.96	1.05	0.852	0.98	0.91	1.05	0.504	1.03	0.96	1.09	0.417
Self-reported general health	1.02	0.99	1.06	0.174	1.00	0.95	1.05	0.961	1.04	0.99	1.08	0.088
Mental health condition	1.11	1.07	1.14	<0.001	1.11	1.04	1.18	0.001	1.10	1.06	1.15	<0.001
Any chronic illness currently treated	1.02	1.00	1.04	0.026	1.02	0.99	1.05	0.218	1.02	1.00	1.05	0.062

SDQT, Strengths and Difficulties Questionnaire Total Score (binary score 0–16 *v*. 17–36); SDQE, Strengths and Difficulties Questionnaire Emotion Score (binary score 0–4 *v*. 5–10.

For all binary variables, odds ratios show the change in odds per year over the study period. Values >1 indicate increasing odds of poor health over time. For scale variables (SDQE, SDQT), the *β* coefficient represents the change in score per year over time. Positive values indicate increasing number of symptoms.

**Table 4.** Odds ratios for changes in mental health and well-being measures over time, by age group and sex. Health Survey for England, 1995–2014

	Total				Males				Females			
	Odds ratio/*β* coefficient	Confidence interval		*p* value	Odds ratio/*β* coefficient	Confidence interval		*p* value	Odds ratio/*β* coefficient	Confidence interval		*p* value
	4–12 years old											
Parent-reported general health	0.965	0.957	0.972	<0.001	0.966	0.956	0.976	<0.001	0.963	0.952	0.973	<0.001
Mental health condition	1.071	1.058	1.083	<0.001	1.076	1.061	1.091	<0.001	1.055	1.030	1.081	<0.001
Long-standing illness	0.977	0.973	0.982	<0.001	0.980	0.974	0.985	<0.001	0.974	0.968	0.981	<0.001
SDQE binary	0.973	0.964	0.982	<0.001	0.978	0.965	0.991	0.001	0.968	0.956	0.980	<0.001
SDQE	−0.024	−0.029	−0.018	<0.001	−0.021	−0.028	−0.014	<0.001	−0.026	−0.034	−0.019	<0.001
SDQT binary	0.979	0.969	0.988	<0.001	0.977	0.965	0.989	<0.001	0.981	0.966	0.996	0.014
SDQT	−0.093	−0.109	−0.078	<0.001	−0.101	−0.124	−0.079	<0.001	−0.085	−0.105	−0.064	<0.001
	13–15 years old											
Self-reported general health	0.973	0.962	0.984	<0.001	0.976	0.960	0.993	0.005	0.970	0.954	0.986	<0.001
Mental health condition	1.084	1.061	1.107	<0.001	1.087	1.059	1.116	<0.001	1.078	1.039	1.118	<0.001
Long-standing illness	0.974	0.966	0.981	<0.001	0.975	0.964	0.985	<0.001	0.972	0.962	0.983	<0.001
SDQE binary	0.978	0.962	0.994	0.006	0.982	0.956	1.008	0.165	0.976	0.956	0.997	0.026
SDQE	−0.022	−0.032	−0.012	<0.001	−0.011	−0.024	0.002	0.112	−0.031	−0.045	−0.016	<0.001
SDQT binary	0.999	0.981	1.018	0.910	0.995	0.971	1.020	0.703	1.003	0.975	1.031	0.861
SDQT	−0.049	−0.078	−0.020	0.001	−0.048	−0.089	−0.006	0.024	−0.053	−0.092	−0.013	0.009
GHQ binary	1.010	0.998	1.022	0.099	1.014	0.995	1.034	0.159	1.009	0.994	1.024	0.250
GHQ	0.002	−0.005	0.009	0.593	−0.001	−0.009	0.008	0.837	0.006	−0.006	0.017	0.324
	16–24 years old											
Self-reported general health	0.987	0.980	0.994	<0.001	0.978	0.967	0.989	<0.001	0.993	0.984	1.002	0.145
Mental health condition	1.087	1.072	1.102	<0.001	1.091	1.068	1.114	<0.001	1.084	1.064	1.103	<0.001
Long-standing illness	0.982	0.977	0.988	<0.001	0.979	0.971	0.987	<0.001	0.984	0.977	0.991	<0.001
GHQ binary	0.989	0.981	0.997	0.005	0.996	0.982	1.009	0.508	0.984	0.975	0.994	0.002
GHQ	−0.016	−0.023	−0.010	<0.001	−0.012	−0.020	−0.003	0.007	−0.021	−0.031	−0.011	<0.001
WEMWBS	−0.109	−0.308	0.090	0.283	−0.212	−0.498	0.075	0.148	−0.042	−0.316	0.232	0.762

SDQT, Strengths and Difficulties Questionnaire Total Score (binary score 0–16 *v*. 17–36); SDQE, Strengths and Difficulties Questionnaire Emotion Score (binary score 0–4 *v*. 5–10); GHQ, General Health Questionnaire (binary score 0–3 *v*. 4–12); WEMWBS, Warwick–Edinburgh Mental Health and Wellbeing Score.

For all binary variables, odds ratios show the change in odds per year over the study period. Values >1 indicate increasing odds of poor health over time. For scale variables (SDQE, SDQT, WEMWBS), the *β* coefficient represents the change in score per year over time. Positive values indicate increasing number of symptoms (SDQT, SDQE) or increasing well-being score (WEMWBS).

### Scotland

Table 2 reported the long-standing mental health conditions increased between 2003 and 2014. This increase was seen in all age groups [4–12 years, 2.6% *v*. 4.8%, odds ratio (OR) 1.05, *p* = 0.004; 13–15 years, 3.0% *v*. 7.8%, OR 1.07, *p* = 0.02; 16–24 years, 3.1% *v*. 9.7%, OR 1.08, *p* < 0.001). This can be interpreted as a 5, 7 and 8% increase per year in the odds of having a long-standing mental health condition in each age group, respectively.

The prevalence of any long-standing condition increased in 16–24 years old (18.1% *v*. 25.6%, OR 1.04, *p* = 0.001). There was no change in general health, GHQ or well-being scores over time in any group. The only trend towards improved health was a significant reduction in high SDQT and SDQE scores among girls aged 4–12 years (SDQT: 7.2% *v*. 4.8%, OR 0.95, *p* = 0.009; SDQE: 9.6% *v*. 6.1%, OR  0.96, *p* = 0.005). No quadratic terms were significant.

In contrast to stable longer term trends, GHQ score increased (worsened) significantly for 16–24 year olds between 2011 and 2014 (OR 0.15, *p* = 0.003). Conversely, there was no change between 2011 and 2014 in the reported prevalence of any long-standing condition in any age group and self-reported general health improved significantly in 16–24 years old over this period (OR 1.18, *p* = 0.007). For all other variables, including long-standing mental health conditions, trends from 2011 to 2014 were broadly similar to the longer term trends (see Appendix Table E).

### Wales

There was no change in any measure among the 4–12 years group. There was a significant increase in high SDQE scores among 13–15 years old (14.4% *v*. 24.2%, OR 1.07, *p* = <0.001) and in young adults reporting treatment for mental health conditions (4.1% *v*. 7.6%, OR 1.11, *p* < 0.001). In contrast, there was no change over time in general health or prevalence of any long-standing condition. There were no significant quadratic terms.

Data from 2011 to 2014 on 16–24 years old showed ongoing increased prevalence of mental health disorders (OR 1.14, *p* = 0.005) and also showed an increased prevalence of any chronic illness (OR 1.08, *p* = 0.007). Recent trends for all other variables were similar to longer term trends, showing a significant increase in SDQE and SDQT for 13–15 years old, and no change in other variables (see Appendix Table E).

### England

There was a consistent increase in the reported prevalence of long-standing mental health conditions across all age and sex groups (range 5.5–9.1% year-on-year increase between 1995 and 2014) (4–12 years: 0.9% *v*. 3.9%, OR 1.070, *p* < 0.001; 13–15 years: 1.1% *v*. 5.4%, OR 1.08, *p* < 0.001; 16–24 years: 0.6% *v*. 5.9%, OR 1.09, *p* < 0.001). Conversely, there was a consistent reduction in the prevalence of any long-standing condition over the same period (4–12 years: 21.5% *v*. 18.2%, OR 0.98, *p* < 0.001; 13–15 years: 23.8% *v*. 18.8%, OR 0.97, *p* < 0.001; 16–24 years: 23.6% *v*. 21.7%, OR 0.98, *p* < 0.001).

The ORs of fair/bad general health decreased among younger participants (4–12 years: OR 0.97, *p* < 0.001; 13–15 years: OR 0.97, *p* < 0.001) but not among young adults (OR 0.99, *p* = 0.07). High SDQT and SDQE scores among young children became less common over time (SDQT: OR 0.98, *p* < 0.001; SDQE: OR 0.97, *p* < 0.001) as did high SDQE scores for 13–15 years old (OR 0.98, *p* = 0.006). There was no change in high SDQT or GHQ scores for 13–15 years old.

The proportion of young adults with high GHQ scores decreased over time (OR 0.99, *p* = 0.005); within the data available (2010–2014), no change was observed in well-being scores in this age group. Quadratic terms were significant for well-being and GHQ among 16–24 years old, suggesting that well-being and psychological distress may have initially improved between 2010 and 2012 before deteriorating from 2012 to 2014 (WEMWBS linear coefficient = 12.0, *p* < 0.001, quadratic coefficient = −0.336, *p* < 0.001; GHQ linear coefficient = 0.81, *p* < 0.001, quadratic coefficient = 1.01, *p* < 0.001). No other quadratic terms were significant.

Consistent with these quadratic findings, data from 2011 to 2014 showed a highly significant decrease in WEMWBS scores for 16–24 years old (*β* coefficient = −0.46, *p* = 0.001). However, there was no significant change in GHQ scores over this period. Recent trends for other variables largely reflected longer term trends, although the prevalence of any long-standing condition was stable rather than decreasing (please see Appendix Table E). Please note that SDQ data were not collected in the HSE after 2011.

### Gender analyses

Analysis of all children aged 4–12 years across the total study period showed that long-standing mental health conditions were more common among boys than girls: England 3.5% *v.* 1.1% (*p* < 0.001), Scotland 5.3% *v.* 1.6% (*p* < 0.001), Wales 1.5% *v.* 0.8% (*p* < 0.001). High SDQT scores were also more common among boys than girls: England 10.6% *v.* 6.9% (*p* < 0.001), Scotland 9.8% *v.* 5.4% (*p* < 0.001), Wales 10.3% *v.* 6.1% (*p* < 0.001).

Gender differences for the 13–15 age group were less consistent. Boys were more likely than girls to report long-standing mental health conditions (England 3.4% *v.* 1.7%, *p* < 0.001; Scotland 5.1% *v.* 2.6%, *p* < 0.001), any long-standing condition (England 24.8% *v.* 21.4%, *p* = <0.001; Scotland 21.2% *v.* 18.3%, *p* = 0.05) and high SDQT (England 9.1% *v.* 7.3%, *p* = 0.002). Conversely, girls were more likely than boys to report high SDQE (England 12.9% *v.* 7.7%, *p* < 0.001), high GHQ (England 13.9% *v.* 7.3%, *p* < 0.001; Scotland 11.9% *v.* 7.0%, *p* < 0.001) and lower well-being (Scotland 50.0% *v.* 52.1%, *p* < 0.001). Sex differences were smaller in Wales in this age group, with the exception of high SDQE scores, which were more common among girls than boys (23.8% *v.* 11.4%, *p* < 0.001).

Among young adults, high GHQ scores were more common, and mean WEMWBS scores were lower, among women than men: [GHQ: England 19.2% *v.* 10.9% (*p* < 0.001), Scotland 19.2% *v.* 11.9% (*p* < 0.001); WEMWBS: England 50.7% *v.* 52.0 (*p* < 0.001), Scotland 49.1% *v.* 50.5 (*p* < 0.001)]. Long-standing mental health conditions were more common among women than men in Wales [6.6% *v.* 3.6% (*p* < 0.001)] but not in England [2.7% *v.* 2.5% (*p* = 0.2)], or Scotland [6.2% *v.* 4.6% (*p* = 0.02)].

## Discussion

To our knowledge, this is the first national-level study to investigate the UK time trends in mental health problems in CYP since 2007. We found a consistent and striking increase in the reported prevalence of a long-standing mental health condition in all three countries. Among CYP aged 4–24 years, the reported prevalence of long-standing mental health conditions increased sixfold between 1995 and 2014 in England and more than doubled between 2003 and 2014 in Scotland. In Wales, where participants were asked to report only conditions for which they were receiving treatment, mental health conditions increased by around half among young adults over the period 2007–2014. Over the same period, reported prevalence of any long-standing condition (of which long-standing mental health conditions are a growing subset) decreased significantly in England. Scales measuring psychological distress showed inconsistent trends, with improvements in SDQT, SDQE and GHQ scores for some age groups in England and Scotland, but deterioration in Welsh SDQE scores for 13–15 years old. Sex differences were as expected from previous studies.

Where direct comparisons were possible, the trends for 2011–2014 were broadly similar to the longer term trends for most variables, although as expected, the recent trends were less statistically significant in many cases. However, there was some evidence of worsening trends in the health of young adults in recent years (increased GHQ scores in Scotland, increased chronic illnesses in Wales and lower well-being scores in England).

There are some limitations to this study arising from the survey content and methodology. The Welsh and Scottish surveys only provide consistent data from 2007 and 2008 onwards, respectively. In comparison, the HSE data provide good coverage over a 19-year period but analysis is limited by some inconsistencies in the content and methodology. For example, GHQ data were not collected in 1996, 2007 and 2013; SDQ data were not collected in 1998, 2000, 2001, 2003, 2009 and/or from 2012 onwards; WEMWBS data were only available from 2010 onwards. While the questions about long-standing illness were included in every survey, the wording and structure has evolved over time. For example, the question changed from ‘*Do you have any long-standing illness, disability or infirmity?*’ (used until 2011) to ‘*Do you have any have any physical or mental health conditions or illnesses lasting or expected to last 12 months or more?*’ (used from 2012 onwards).

However, there was no evidence from the quadratic analyses, the separate analyses of recent trends or from Fig. 1 that this wording change had any significant impact on the trend to increased mental health disorders.

Another significant limitation to interpreting these findings is the lack of data on the impact or nature of a mental health condition. Validated items on impact are available as an additional part of the SDQ and could usefully be included in future surveys. Regarding the nature of mental health conditions, it is not possible to investigate how self-/parents’ understanding of mental health conditions compares to clinical definitions or whether these understandings have changed over time. However, there is little evidence to suggest that increases in developmental or behavioural disorders can explain the greater reported prevalence of long-standing mental health conditions, especially as the increases are more pronounced among young adults. Lastly, weighting was not available for adult participants of the HSE before 2002, and weighting methodology has subsequently changed over time. However, the high level of agreement between unweighted and weighted analyses, where direct comparison is possible, provides some reassurance that lack of weighting information and/or changing composition of the sample over time are unlikely to affect our findings significantly. Similarly, response rates are reasonably consistent over time.

Our findings are consistent with the previous evidence of increasing rates of mental health diagnoses among CYP in recent decades across many high-income countries. Collishaw (2015) identified several possible factors that may have contributed to higher rates of mental health diagnoses, including increased help-seeking by parents and young people, improved screening and clinical recognition in schools and primary care, medicalisation of feelings and behaviours previously considered as normal, as well as a possible genuine increase in pathology (Collishaw, 2015). Green *et al*. (2005) reported a discrepancy between rates of diagnosis based on a clinical interview (10%) and self-report of mental health diagnosis (1–3%) (Green *et al*., 2005). It is possible, therefore, that much of the reported increase in mental health conditions may simply reflect narrowing of the gap between problems that exist and problems that are reported – for example, due to increased awareness of mental health problems, and/or reduced stigma in acknowledging and seeking help for problems. Although this study could not investigate the impact of specific programmes, it is possible that initiatives such as Targeted Mental Health in Schools (TAMHS) (National_Child_and-Maternal_Health_Intelligence_Network) and Improving Access to Psychological Therapies for Children and Young People (CYP IAPT) (NHS_England) in England may have contributed to greater awareness of mental health issues among professionals in schools and primary care, as well as among parents and CYP themselves.

Our findings differ from previous studies that reported an increase in emotional distress, and decrease in emotional well-being in adolescents, alongside a widening gender gap between girls and boys. For example, Fink *et al*. (2015) reported an increase in emotional distress in 11–13 years old girls in England between 2009 and 2014, and the Health Related Behaviour in School-Age Children (HBSC) study reported a trend to decreased well-being of 15-year-old girls in England, 2002–2014 (Brooks *et al*., 2015; Fink *et al*., 2015) Similarly, analyses of data from the Longitudinal Study of Young People in England (LSYPE) showed that mean GHQ scores were slightly worse in 2014 than in 2005 and that the gap between girls and boys had widened slightly over this period (Lessof *et al*., 2016). In contrast, we did not find any increase in mental health problems among early adolescent girls in England or Scotland, and SDQE scores decreased significantly among 13–15 years old girls in England. With the exception of 4–12 years old in Scotland (where girls had lower SDQT than boys and showed a greater decrease over time), we found no significant difference in trends between males and females for any country or age group. However, we note that our analyses are over a longer time period than many comparable studies, especially in England, so their findings are not directly comparable. As noted above, sensitivity analyses of the most recent four surveys (2011–2014) show that trends during this period are broadly consistent with longer term trends, but there was some evidence of worsening trends in the health of young adults.

### Clinical, policy and research implications

Although the year-on-year changes are relatively small in many cases, the sustained trends over many years have led to significant changes in population-level prevalence of long-standing mental health conditions among CYP in the UK (as highlighted in Fig. 1), with significant implications for health services. Parental or CYP perceptions of a long-standing mental health condition may have an important impact on clinical consultations and the success of treatment for both mental and physical health problems. Most UK clinicians have responsibility for some patients aged 0–24 years and should be aware of the increasing need to consider mental health diagnoses in this age group. For mental health professionals, the differing trends between reported long-standing mental health conditions and questionnaire scores suggest that the use and interpretation of such scores in assessing patients should continue to evolve. Further investigation is needed, but it is possible that an increasing range of mental health issues is not adequately captured by existing questionnaires.

Although our data do not allow us to attribute causality, or to separate the effect of multiple interacting influences on mental health, our findings are nevertheless relevant to several current policy debates. The growing burden of mental health problems faced by CYP, both in absolute terms, and as a proportion of all long-standing health conditions (mental and physical) reinforces the growing demand for CAMHS services, which is already posing an important challenge for UK health services and policymakers. Even positive initiatives such as CYP IAPT and increased funding for CAMHS are unlikely to address the scale of unmet need for CAMHS services that has been widely reported elsewhere and is reinforced in our data. However, as many of the determinants and potential solutions to mental health problems in CYP lie beyond healthcare (Ford *et al*., 2007; Newlove-Delgado *et al*., 2015), multi-sectoral initiatives will also be needed to address wider determinants of mental health problems, ranging from poverty and impact of cuts to benefits and youth services, to modern societal pressures of cyberbullying and early sexualisation.

Our findings also highlight some interesting research gaps. First, better data are clearly needed to inform and evaluate the national CAMHS strategy, and so improve short- and long-term outcomes of mental health problems in CYP. Second, the apparent paradox of increasing rates of long-standing mental health conditions but no consistent increase in psychological distress deserves further investigation. One possibility is that there has been a change in the distribution of common symptoms such as sadness and anxiety within society. For example, Lessof *et al*. reported a trend towards higher mean GHQ scores, despite no change in the proportion of people reporting above-threshold scores (Lessof *et al*., 2016). Our data replicate this finding for young women in Scotland and young men in England, with a significant increase in mean GHQ score over time, but no increase in the proportion with above-threshold scores. However, it is not clear whether this reflects a genuine change in the distribution of mental health symptoms, or just that the GHQ total score is a more sensitive measure of change. Interestingly, the reverse pattern is seen in English 13–15 years old for all SDQT scores and female SDQE score (i.e. a significant decrease in mean overall score, but no decrease in the proportion with above-threshold score). It is also possible that the threshold for self-/parent report of a mental health condition has diverged over time from clinical thresholds, which may explain some of the discrepancy we found between trends for self-/parent reports of a long-standing mental health condition and above-threshold questionnaire scores. The detailed national study of mental health problems in CYP that is currently underway may allow more detailed investigation of this issue.

Lastly, trends in socio-economic inequalities were beyond the scope of this study, but deserve more detailed investigation in the future. LSYPE data showed that, while trends towards increased GHQ scores were particularly marked in CYP from single-parent and reconstituted families, other measures of psychological distress now appeared to be more prevalent among CYP living in more affluent households (Lessof *et al*., 2016).

## Conclusions

There has been a striking increase in the reported prevalence of long-standing mental health conditions among CYP in England, Scotland and Wales in recent years, over a period of progressive improvement in many physical and general health measures. We report an apparent discrepancy between increases in reported prevalence of long-standing mental health disorders and relatively little change in scores on questionnaires relating to psychological distress and emotional well-being. This discrepancy deserves further investigation.

Our findings reinforce the need to consider what constitutes a mental health problem for young people and to continue to prioritise resources to help young people and their families overcome difficulties they are experiencing. While policy initiatives to promote access to CAMHS services and parity of esteem for mental and physical health are clearly needed, they may be ineffective unless they are adequately funded and accompanied by parallel measures to promote mental health and well-being in schools and wider society.

## Supplementary material

For supplementary material accompanying this paper visit https://doi.org/10.1017/S0033291718001757. click here to view supplementary material
