# Supplementary material for: Mental health and well-being trends among children and young people in the UK, 1995–2014: analysis of repeated cross-sectional national health surveys
Source: Psychol Med. 2018 Sep 11;49(8):1275–85. doi: 10.1017/S0033291718001757 (PMC6518382; doi:10.1017/S0033291718001757)

**Supplemental material - contents**

Appendix Table A. Number of participants by sex, age, and country. . ………….......... 2

Appendix Table B. Wording of the questions and responses by country and age……….3

Appendix Table C. Prevalence of each health measure by country, age, sex,

and year …………………………………………………………………………………. 7

Appendix Table D. Sensitivity analyses comparing regression models with

and without weighting.………………………………………………………………… 19

Appendix Table E. Sensitivity analyses showing regression models of recent trends

2011-14…………………………………………………………………………………. 23

**Appendix Table A. Number of participants by country, year, and sex.**

**Appendix Table B. Wording of questions and responses by country and age.**

In each case, wording is taken from the most recent relevant survey.

1. Self/parent-reported health:

*Scotland/England (all ages). How is your/your child’s health in general? Would you say it was very good, good, fair, bad, or very bad?*

*Wales (adults). In general would you say your health is Excellent, Very good, good, fair, poor*

*Wales (4-12). How is this child’s health in general? Would you say it was…Very good, good, fair, bad, very bad*

*Wales (13-15): How is your health in general? Would you say it was Very good, good, fair, bad, very bad*

1. Presence of any long-standing condition: Yes/No. (England and Scotland only)

Presence of any currently treated chronic illness: Yes/no. (Wales only)

1. Presence of any long-standing mental health condition: Yes/No. (England and Scotland only)

Presence of any currently treated mental health problem: Yes/no (Wales only)

*Scotland/England : Do you/your child have a physical or mental health condition or illness lasting, or expected to last 12 months or more? (yes/no).*

*Scotland: If responds yes, what (else) is the matter with you (up to six long-standing illnesses recorded).*

*One option is Mental illness/anxiety/depression/nerves (nes)*

*England: If responds yes, do any of your conditions or illnesses affect you in any of the following areas?*

*One option is ‘mental health’*

*Wales (4-12): Is this child currently being treated, by a doctor, consultant or specialist, for any of these? Options include ‘Anxiety, depression or mental illness’*

*Wales (13-15): Are you currently being treated, by a doctor, consultant or specialist, for any of these?Options include ‘Anxiety, depression or mental illness’*

*Wales (adults): ‘Are you being currently treated for any of these illnesses? Options include depression, anxiety, another mental illness.*

1. Strengths and Difficulties Questionnaire Total difficulties (SDQT) score:[26]

Both the total score and a dichotomised score of 0-16 vs. 17-40 were analysed.

1. Strengths and Difficulties Questionnaire Emotional difficulties (SDQE) score:[26] The full score and a dichotomised score of 0-4 vs. 5-10 were analysed. We analysed the SDQE score separately to the total SDQ as previous studies have raised specific concerns about increasing rates of emotional distress, especially among adolescent girls.[9] Detailed analysis of other subscales was beyond the scope of this study For both SDQT and SDQE, we used thresholds previously published for analysis of parent-reported SDQ scores for children aged 4-17.[27] Individuals who scored above the described threshold were considered to have screened positive for significant mental health symptoms.

***Emotional problems scale***

*ITEM 3: Often complains of headaches…*

*ITEM 8: Many worries*

*ITEM 13: Often unhappy, downhearted*

*ITEM 16: Nervous or clingy in new situations…*

*ITEM 24: Many fears, easily scared*

***Conduct problems Scale***

*ITEM 5: Often has temper tantrums or hot tempers*

*ITEM 7: Generally obedient…*

*ITEM 12: Often fights with other children*

*ITEM 18: Often lies or cheats*

*ITEM 22: Steals from home, school or elsewhere*

***Hyperactivity scale***

*ITEM 2: Restless, overactive…*

*ITEM 10: Constantly fidgeting or squirming*

*ITEM 15: Easily distracted, concentration wanders*

*ITEM 21: Thinks things out before acting*

*ITEM 25: Sees tasks through to the end.*

***Peer problems scale***

*ITEM 6: Rather solitary, tends to play alone*

*ITEM 11: Has at least one good friend*

*ITEM 14: Generally liked by other children*

*ITEM 19: Picked on or bullied by other children…*

*ITEM 23: Gets on better with adults than with other children*

Each item is scores from 0= not true, 1= somewhat true, 2= certainly true.

Total difficulties score greater than or equal to 17/40 is categorised as reflecting significant psychological distress.

Emotional problems score greater than or equal to 5/10 is categorised as reflecting significant emotional problems.

For further details see http://www.sdqinfo.com/py/sdqinfo/b3.py?language=Englishqz(UK)

1. General Health Questionnaire (GHQ) score [28]: The total score and a dichotomised score (0-3 vs. 4-12) were analysed, following previous analysis of GHQ scores in the Health Survey for England.[29]

HAVE YOU RECENTLY:

*Been able to concentrate on whatever you’re doing?*

*Lost much sleep over worry?*

*Felt you were playing a useful part in things?*

*Felt capable of making decisions about things?*

*Felt constantly under strain?*

*Felt you couldn’t overcome your difficulties?*

*Been able to enjoy your normal day-to-day activities?*

*Been able to face up to your problems?*

*Been feeling unhappy and depressed?*

*Been losing confidence in yourself?*

*Been thinking of yourself as a worthless person?*

*Been feeling reasonably happy, all things considered?*

For each question, respondents are asked to tick one of four boxes

Better than Usual, Same as usual score =0

Less than usual, Much less than usual. Score = 1.

Scores are summed to give a total score from 0-12.

Scores greater than or equal to 4 were categorised as reflecting significant psychological distress.

General Health Questionnaire (GHQ-12) ©David Goldberg 1978.

1. Warwick-Edinburgh Mental Wellbeing Scores (WEMWBS)[30]. No standard threshold have been published, so only the total score was analysed.

*Below are some statements about feelings and thoughts.*

*Please tick the box that best describes your experience of each over the last 2 weeks*

*I’ve been feeling optimistic about the future*

*I’ve been feeling useful*

*I’ve been feeling relaxed*

*I’ve been feeling interested in other people*

*I’ve had energy to spare*

*I’ve been dealing with problems well*

*I’ve been thinking clearly*

*I’ve been feeling good about myself*

*I’ve been feeling close to other people*

*I’ve been feeling confident*

*I’ve been able to make up my own mind*

*about things*

*I’ve been feeling loved*

*I’ve been interested in new things*

*I’ve been feeling cheerful*

*None of the time = 1*

*Rarely = 2*

*Some of the time = 3*

*Often = 4*

*All of the time -= 5*

Total score is calculated from 14-70.

© NHS Health Scotland, University of Warwick and University of Edinburgh,

2006, all rights reserved.

**Appendix Table C. Prevalence of each health measures by country, age, sex, and year**

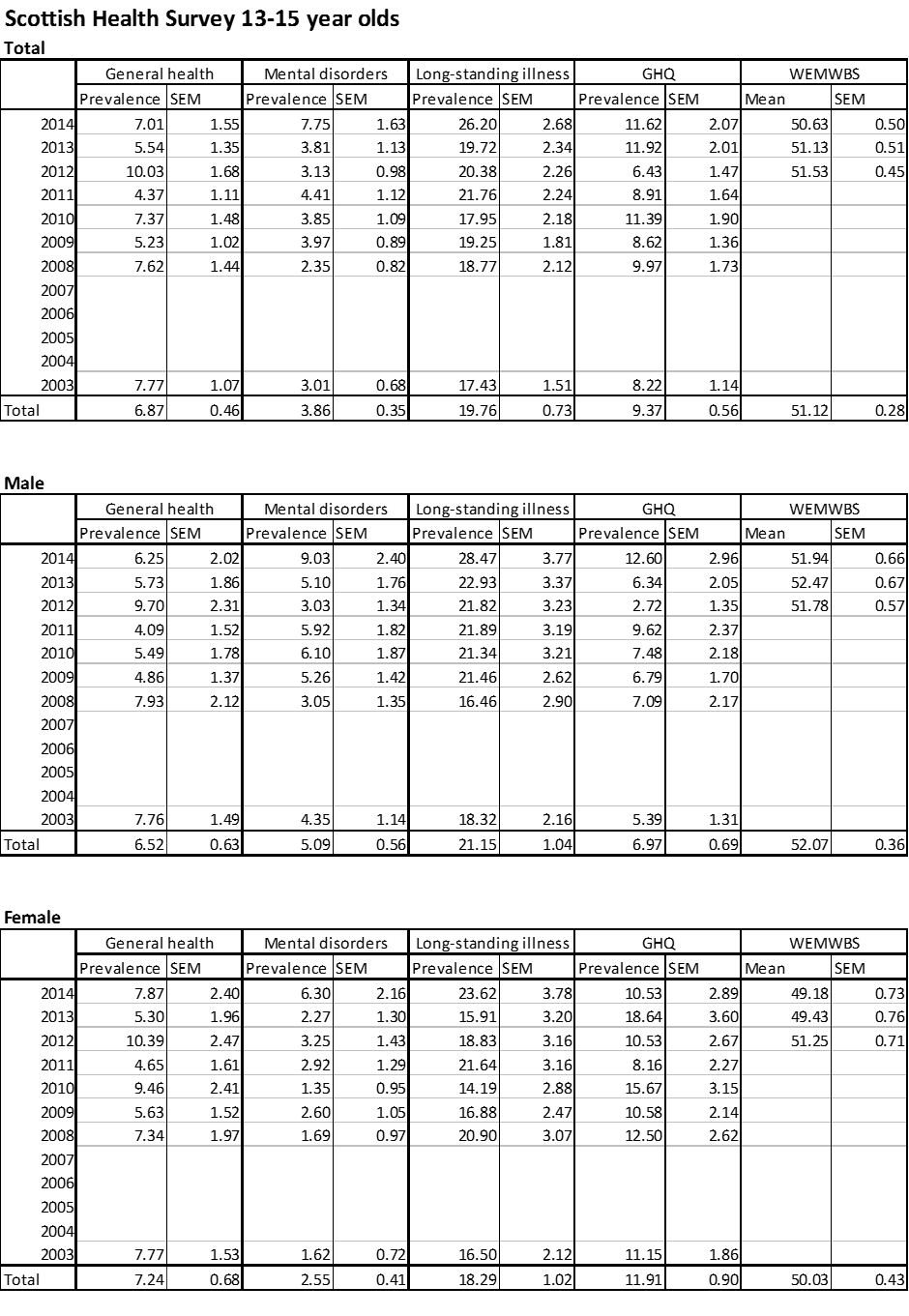

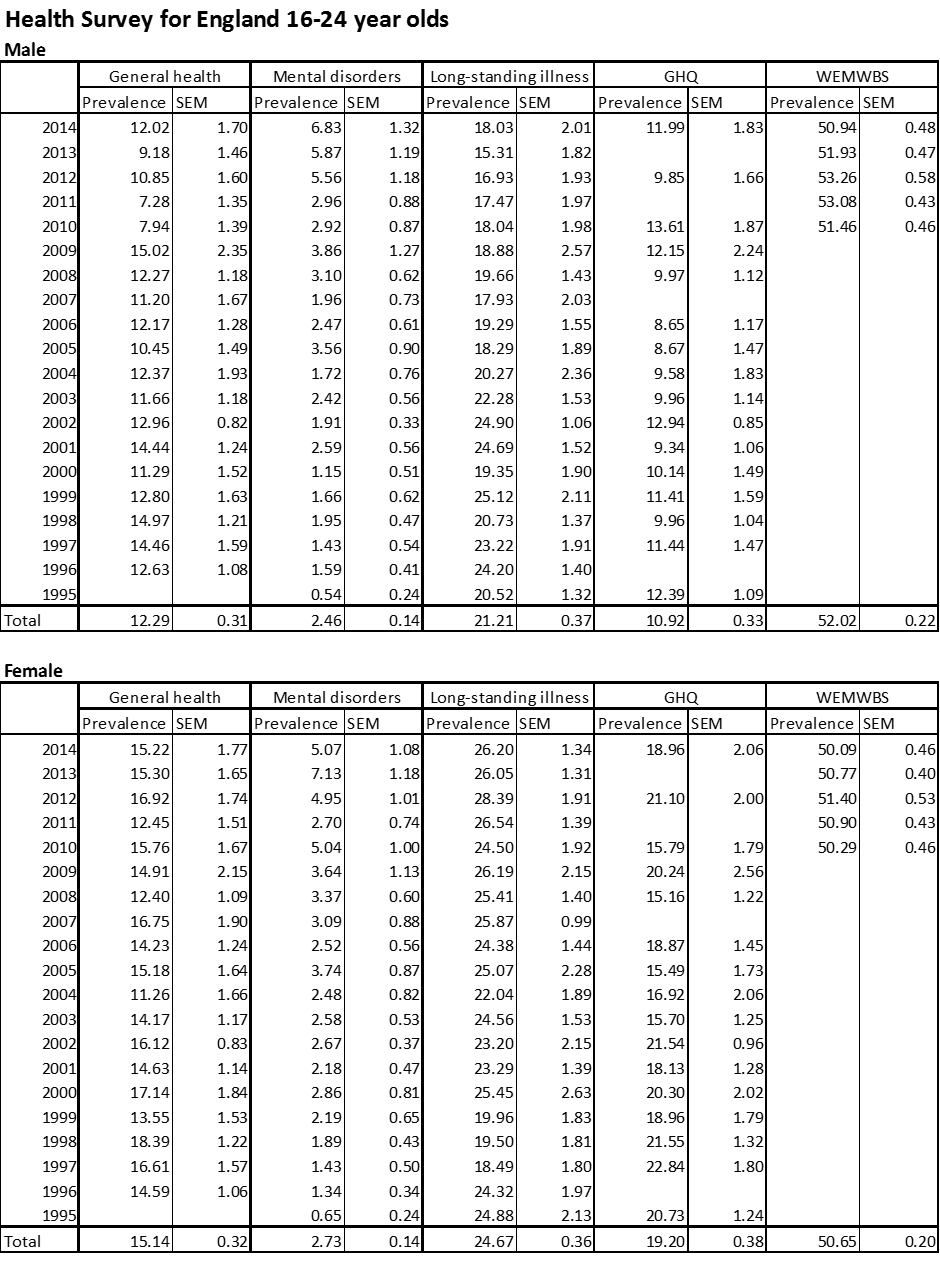

**Appendix Table D. Sensitivity analyses comparing regression models with and without weighting.**

**Sensitivity analyses comparing regression models with and without weighting.**

**Welsh Health Survey.**

**Sensitivity analyses comparing regression models with and without weighting.**

**Scottish Health Survey.**

**Sensitivity analyses comparing regression models with and without weighting.**

**Health Survey for England, 2002-2014.**

**Appendix Table E. Sensitivity analyses showing regression models of recent trends, 2011-14**

**Odds ratios for changes in mental health and well-being measures over time, by age group. Scottish Health Survey, 2011-2014.**


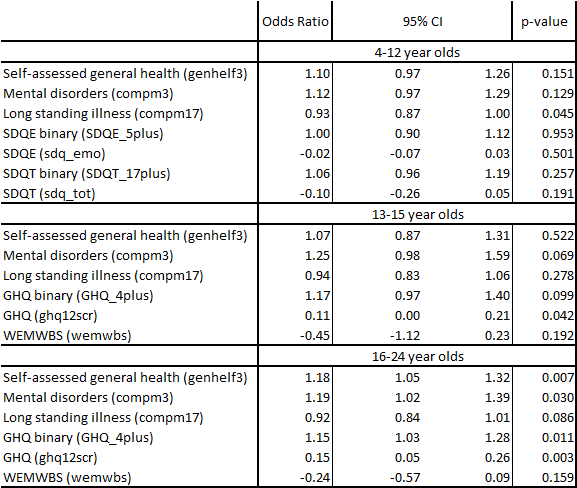


**Odds ratios for changes in mental health and well-being measures over time, by age group. Welsh Health Survey, 2011-2014.**


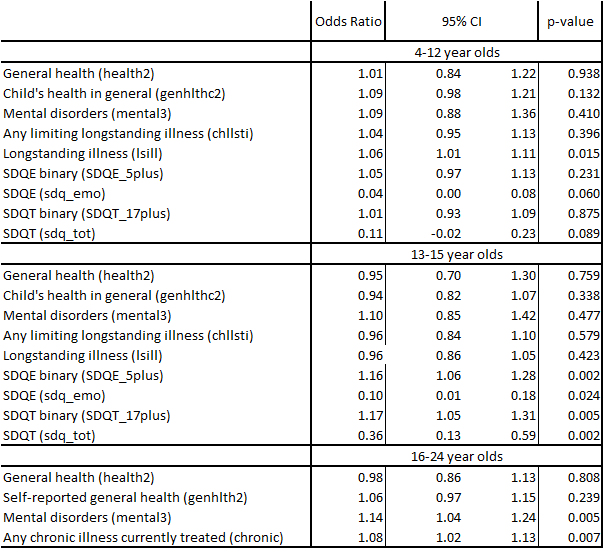


**Odds ratios for changes in mental health and well-being measures over time, by age group. Health Survey for England, 2011-2014.**


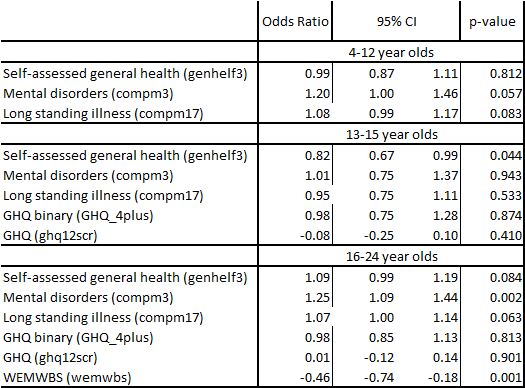

Supplement: Supplementary file 1 [file S0033291718001757sup001.docx]
